# Alterations in Cervical Nerve Root Function during Different Sitting Positions in Adults with and without Forward Head Posture: A Cross-Sectional Study

**DOI:** 10.3390/jcm12051780

**Published:** 2023-02-23

**Authors:** Maryam Kamel, Ibrahim M. Moustafa, Meeyoung Kim, Paul A. Oakley, Deed E. Harrison

**Affiliations:** 1 Department of Physiotherapy, College of Health Sciences, University of Sharjah, Sharjah 27272, United Arab Emirates; 2 Neuromusculoskeletal Rehabilitation Research Group, Research Institute of Medical and Health Sciences, University of Sharjah, Sharjah 27272, United Arab Emirates; 3 CBP Nonprofit (A Spine Research Foundation), Eagle, ID 83616, USA; 4 Independent Researcher, Newmarket, ON L3Y 8Y8, Canada; 5 Kinesiology and Health Sciences, York University, Toronto, ON M3J 1P3, Canada

**Keywords:** sitting, cervical spine, posture, evoked potentials, radiculopathy

## Abstract

The current study aimed to determine whether participants with and without forward head posture (FHP) would respond differently in cervical nerve root function to various sitting positions. We measured peak-to-peak dermatomal somatosensory-evoked potentials (DSSEPs) in 30 participants with FHP and in 30 participants matched for age, sex, and body mass index (BMI) with normal head posture (NHP), defined as having a craniovertebral angle (CVA) >55°. Additional inclusion criteria for recruitment were individuals between the ages of 18 and 28 who were in good health and had no musculoskeletal pain. All 60 participants underwent C6, C7, and C8 DSSEPs evaluation. The measurements were taken in three positions: erect sitting, slouched sitting, and supine. We identified statistically significant differences in the cervical nerve root function in all postures between the NHP and FHP groups (*p* < 0.001), indicating that the FHP and NHP reacted differently in different positions. No significant differences between groups for the DSSEPs were identified for the supine position (*p* > 0.05), in contrast to the erect and slouched sitting positions, which showed a significant difference in nerve root function between the NHP and FHP (*p* < 0.001). The NHP group results were consistent with the prior literature and had the greatest DSSEP peaks when in the upright position. However, the participants in the FHP group demonstrated the largest peak-to-peak amplitude of DSSEPs while in the slouched position as opposed to an erect position. The optimal sitting posture for cervical nerve root function may be dependent upon the underlying CVA of a person, however, further research is needed to corroborate these findings.

## 1. Introduction

Sustained sitting postures and the related load on the cervical spine are important contributors to the high prevalence of neck pain [1]. Prolonged hours of sitting have shown a large incidence of pain in the head, neck, and shoulder region [2,3,4,5]. The optimum sitting position is generally accepted to be a maintained and erect upright spinal position [6]. As described by physiotherapists, an optimal sitting posture is the position with the least amount of muscle activation and the most relaxed and comfortable posture for the entire spine [7,8]. Presumptuously, any deviations away from this erect sitting posture is causative of pain and discomfort [9].

One issue regarding these mechanical ideologies, and popular clinical assumptions supporting the erect sitting posture, is that there is no evidence-based agreement on the optimal sitting posture, especially regarding the neck region [9,10,11,12]. Several studies support the erect sitting as an optimal posture for the head and neck region as mechanically, a more upright sitting posture reduces forward head translation and cervical flexion positions [11,13]. Reducing forward head posture (FHP) and cervical flexion posture by changes in sitting position modification has a direct influence on neck flexor and extensor muscles [14,15].

An issue that is not typically addressed when assessing sitting posture is the presence of pre-existing spinal misalignment or poor postures. FHP is a common poor posture that is associated with a greater load transmitted to the neck [16,17], greater muscle activation and fatigue [18], lower endurance of the deep neck extensors and flexors [19], as well as substantial effects on the biomechanics of the nervous system by causing unfavorable mechanical strain [20,21], which causes the blood vessels to constrict [22] and the nerve root sleeves to unfold and become taut, predisposing individuals to altered or inefficient neurophysiological symptoms [23,24]. Accordingly, we believe the combined effects of sitting with a pre-existing FHP may likely exacerbate any overstraining of the spine and soft tissues, including any neurophysiological effects.

Those with FHP have been demonstrated to exhibit abnormal sensorimotor control as well as autonomic nervous system dysfunction as compared to persons without FHP [23]. It has also been shown that the therapeutic correction of FHP and cervical lordosis aids in the improvement of sensorimotor control [24]. It is unknown, however, whether immediate changes in sitting posture have the potential to create alterations in neurophysiologic parameters and how these may differ between persons with and without pre-existing FHP. Consequently, the current study aimed to determine whether participants with and without FHP would respond differently in terms of dermatomal somatosensory-evoked potentials (DSSEPs) to variations in sitting positions versus a supine posture. In terms of neurophysiological outcomes, dermatomal somatosensory-evoked potentials (DSSEPs) are methods for recording cerebral-evoked reactions to the stimulation of specific regions innervated by single nerve roots, with the goal of supplying pure sensory input to the central nervous system through individual spinal segments to provide reliable information about segmental nerve root function [25].

## 2. Methods

Sixty (60) healthy participants voluntarily agreed to participate in this cross-sectional study. These two groups were parallel matched in age, body mass index (BMI), and sex. Ethics approval was obtained from University of Sharjah Research Ethics Committee in April 2021 REC-19-10-31-02-S. Following Ethics Committee approval, participant recruitment was from April 2021 to August 2022. Informed consent was obtained from all participants prior to the experiment according to relevant guidelines and regulations.

Participants in the NHP group were allocated as closely as possible to match those in the FHP group. Their age was accepted if it was within 2 years apart, the BMI was likewise matched if their BMI varied within 1–2 points. All participants were screened prior to enrollment into the study. The exclusion criteria were as follows: any inflammatory joint disease, systemic pathologies, previous history of musculoskeletal injuries or surgery, spinal disorders, extremity pathologies, or musculoskeletal pain 3 months prior to the study. Exclusion criteria information was obtained through each participant’s medical records. Exclusions were further made of participants during the analysis of the peripheral nerve folly (N9), as detailed below in the neurophysiological assessment section. Participants with an abnormal N9 were excluded. DSSEP peaks follow a normal known structure, and any abnormalities appear clearly. The N9 DSSEP peak represents the afferent signals coming from the brachial plexus. Therefore, any participants with an abnormal N9 were excluded, to remove any possibility of unrelated peripheral factors.

**Figure 1:**
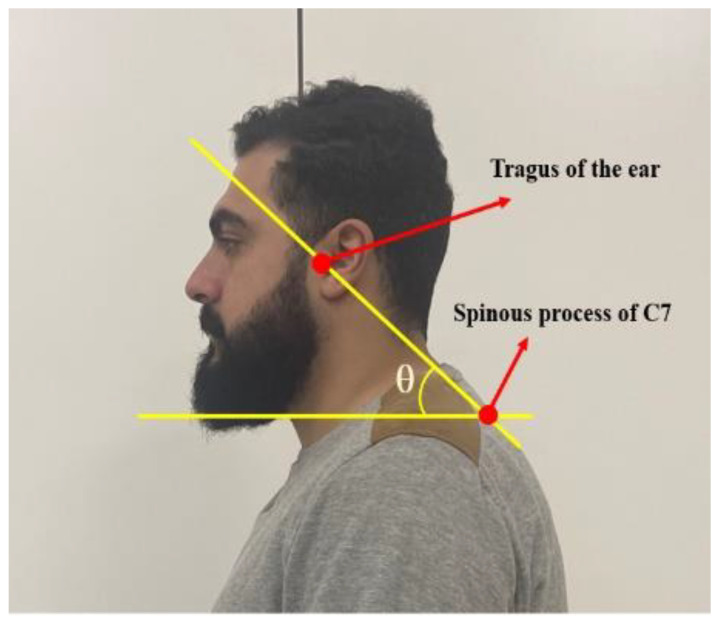
Measurement of the cranio-vertebral angle (CVA). The CVA is represented as the angle above. It is formed by the line connecting two adhesive markers placed at the tragus of the ear and the C7 spinous process; then, this line is assessed relative to a horizontal line drawn through the C7 marker. The angle θ represents the CVA.

The study inclusion criteria for recruitment were any individual between the ages of 18 and 28 who was in good health and had no musculoskeletal pain. The specific allocation of participants to either the FHP or the NHP group was determined by the photogrammetric craniovertebral angle (CVA) of each person [26]. Participants having a CVA below 50° were assigned to the FHP group while participants having a CVA greater than 55° (considered as the cut-off for non-FHP) were assigned to the normal head posture (NHP) group. The CVA measurement method is shown in Figure 1.

### 2.1. Procedures

#### 2.1.1. Evaluation of CVA

The CVA has a high inter-rater and intra-rater reliability in the assessment of FHP [27]. CVA is defined by the angle measured between the horizontal line bisecting the spinous process of C7 and the diagonal line going from the C7 spinous process to the tragus of the ear. As mentioned, we considered a CVA less than 50° to be the threshold for our FHP as this is related to an increased FHP, and FHP is related to increased disability [27].

We followed the published protocol of Falla et al. for the CVA assessment [28]: neutral lateral photos of every participant were taken. Each participant was instructed to sit up in a neutral and comfortable position on a chair and look forward. The photograph was then assessed for the CVA. A digital single-lens reflex camera was placed on a tripod 0.8 m away from the participant. The camera was perpendicular to the sagittal plane of the individuals’ seated position at a height that corresponded with the seventh cervical vertebra of each seated participant. Florescent adhesive markers were used to identify the tragus and the C7 spinous process for the photos. All participants assumed and were assessed in the following three positions for the experiment.

#### 2.1.2. Positions

All 60 participants in their respective group underwent C6, C7 and C8 dermatomal somatosensory-evoked potentials (DSSEPs). For each of the cervical nerves (C6, C7 and C8), measurements were taken in three positions for each participant:

- Supine position (which acted as a reference for DSSEPs measurement);
- After assuming the erect sitting posture for 30 min;
- After assuming the slouched sitting posture for 30 min.

##### Erect Sitting Position

**Figure 2:**
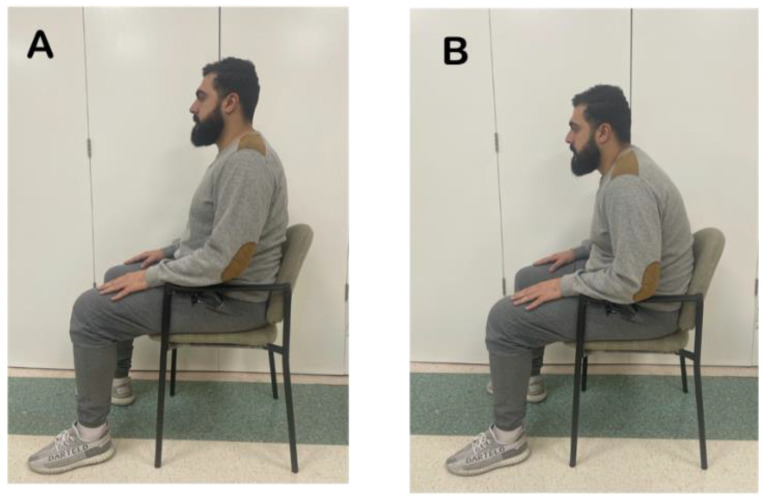
Sitting Positions. (**A**): Erect sitting, (**B**): Slouched sitting.

As shown in Figure 2, the participants sat on a chair supporting their back. Their hips and knees were positioned at a 90° angle, where the base of support was perpendicular to the chair. The arms were rested on the armrest and the spine was assumed in a ‘neutral upright position’ (i.e., neutral kyphosis and lordosis angles); therefore, achieving a slight anterior rotation of the pelvis. Participants were instructed to look forward at a stationary point straight ahead of them.

##### Slouched Sitting Position

Participants sat on the same chair with their back supported and were instructed to relax their thoracolumbar spine to produce a hyperkyphotic angle at the thorax and a straightened lordotic curve at the lumbar region, as shown in Figure 2. This causes a posterior tilt of the pelvis, hyper-kyphosis of the thoracic spine, and a pronounced forward head posture.

##### Supine Position

Participants were instructed to lay back on a flat plinth with the arms in an extended anatomical position. The hip angle was at 180 degrees [29]. The head was supported by a pillow to prevent interference or movement of the electrode placements [30].

### 2.2. Neurophysiological Outcome Measures

#### DSSEPs

**Figure 3:**
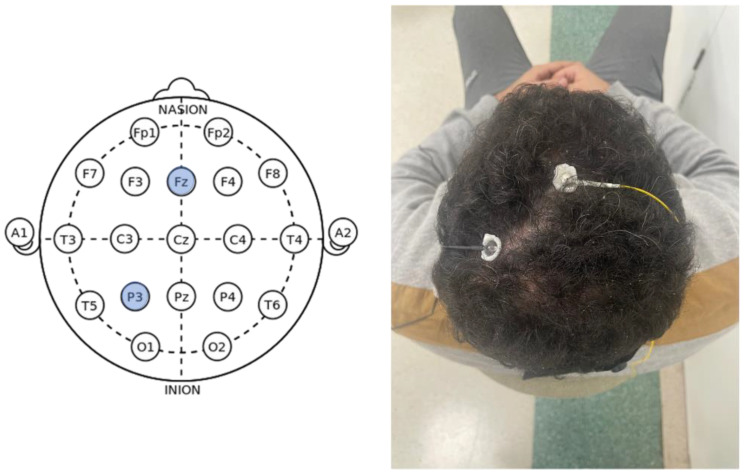
**Left**: Electrode placement following the 10–20 international EEG system; **Right**: Reference and recording electrode placements.

Neurophysiological findings for C6, C7, and C8 were measured in this study as the peak-to-peak amplitude of DSSEPs. An electromyography device (Neuropack S1 MEB-9400K, Nihon Koden, Tokyo, Japan) was used for these neurophysiological assessments. DSSEPs were stimulated with a continuous electrical pulse wave (0.5 ms) at 3 Hz, delivered by three standard surface gel electrodes (20 mm) placed over the respective cervical dermatome; a reference electrode, a recording electrode, and a grounding electrode were used. The stimulation intensity used was above each participant’s perception threshold. All participants initially assumed a relaxed supine position where they were instructed to lay quietly and with eyes closed during the procedure. After parting the hair and using alcohol to prepare the skin, Nuprep gel and Ag–AgCl disc recording electrodes (10 mm with 60 inch lead wires) were fixed with Elefix paste to the scalp (Nihon Kohden, Tokyo, Japan) (Figure 3 shows the electrode placement). The grounding electrode was attached to a strap, which was secured around the forearm. The impedance of all three electrodes was kept below 5 kΩ for an even reading. Three recordings were done for each of the dermatomes stimulated (C6, C7, and C8). The stimulation points were radial forearm 1 inch above the wrist, the middle of the palm right below the middle finger, and the ulnar side of the palm, respectively.

### 2.3. Statistical Analysis

#### 2.3.1. Sample Size

Estimates of mean and standard deviations (SD) from a pilot study of 10 individuals who received the same program were collected to determine the required number of participants in this study. The mean differences and SD of the peak-to-peak amplitude of DSSEPs for different levels C6, 7, and 8 for the different sitting postures: supine, erect and slouched, were: C6: −0.1 (SD 0.3), −0.17 (SD 01.2), −0.86 (SD 0.6); C7: −0.07 (SD 0.9), −0.6 (SD 0.9), −1.6 (SD 1.00); and C8: −0.1 (SD 0.4), −0.9 (SD 0.8), −1.6 (SD 0.9), respectively. The sample size was calculated independently for each of the key outcomes using a Bonferroni correction to adjust the significance level. The greatest sample size value was then used as the trial’s final sample size. Given a statistical power of 80%, the current investigation required at least 25 individuals in each group. To accommodate for probable dropouts, the sample size was increased by 20%.

#### 2.3.2. Data Analysis

Levene’s test of equality of error variances was used to determine the normality distribution of the dataset at 95% confidence interval and *p*-value < 0.05. The dataset had a 2 × 3 factorial design. Descriptive statistics (mean ± SD) were summarized for each position and cervical nerve root. The unpaired *t*-test for continuous variables was used to compare the means and determine the significance of the interaction between the nerve roots in the different sitting positions. A two-way analysis of variance (ANOVA) was then used to test the relationships between the head posture (NHP vs. FHP) and sitting position (supine, slouched, and erect) on the cervical nerve roots (C6, C7, and C8). A *p*-value of 0.05 or less was considered a statistically significant difference in the dataset. Following that, the Tukey honestly significant difference (HSD) post hoc tests were used. SPSS version 29.0 software was used for analyzing data (SPSS Inc., Chicago, IL, USA).

## 3. Results

**Figure 4:**
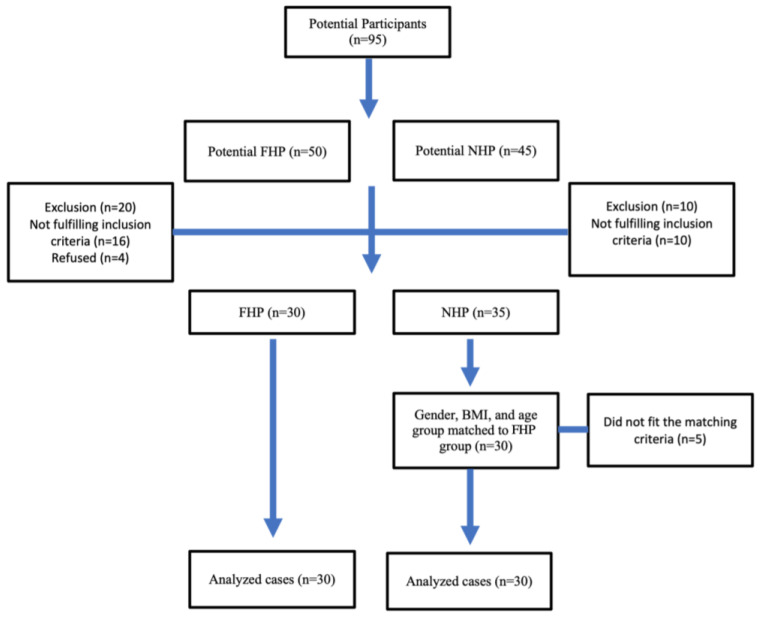
Participant flowchart.

Ninety-five potential participants were initially screened. Thirty participants with FHP and thirty age-, BMI-, and sex-matched controls without FHP were recruited for the NHP group. Figure 4 shows the participant flow chart with numbers excluded and reasons why. Descriptive data for the baseline participant demographics are presented in Table 1. No statistically significant differences between the NHP and the FHP group were found at baseline for their demographic variables. Table 1 shows the mean and distribution of CVA for both groups.

While the number of females in both groups was nearly double that of males, adding sex as a fixed variable to our statistical models in this study did not produce any difference in the outcome findings. A two-way analysis of variance (two-way ANOVA) identified significant head posture × sitting position effects on the outcome of peak-to-peak amplitudes of the cervical nerve roots C6, C7 and C8. Results showed a statistically significant interaction between the head posture and sitting position (F = 32.867) (*p* < 0.001), (F = 38.926) (*p* < 0.001), (F = 40.348) (*p* < 0.001) for C6, C7 and C8, respectively. Table 2, Table 3 and Table 4 presents these data.

**Figure 5:**
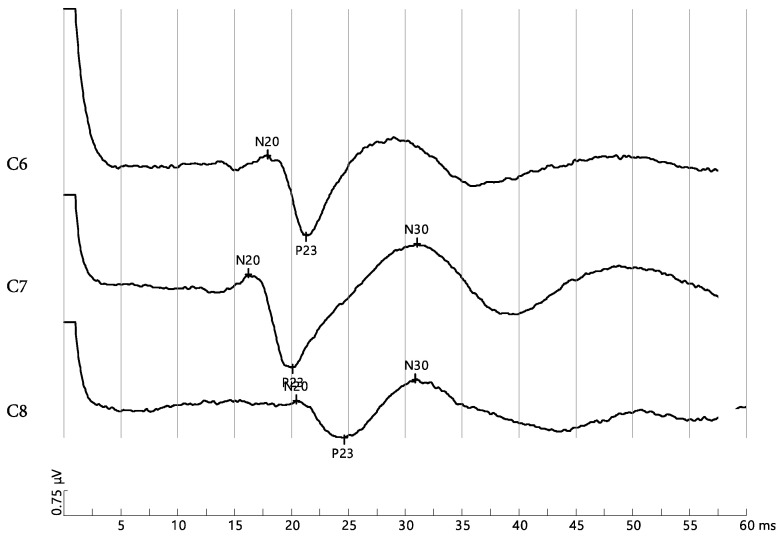
Short latency somatosensory-evoked potential of a normal head posture (NHP) participant before prolonged slouched sitting for C6, C7 and C8. The amplitudes measured between N20 and P23 are: 2.45 μV, 2.8 μV, and 1.14 μV, respectively.

**Figure 6:**
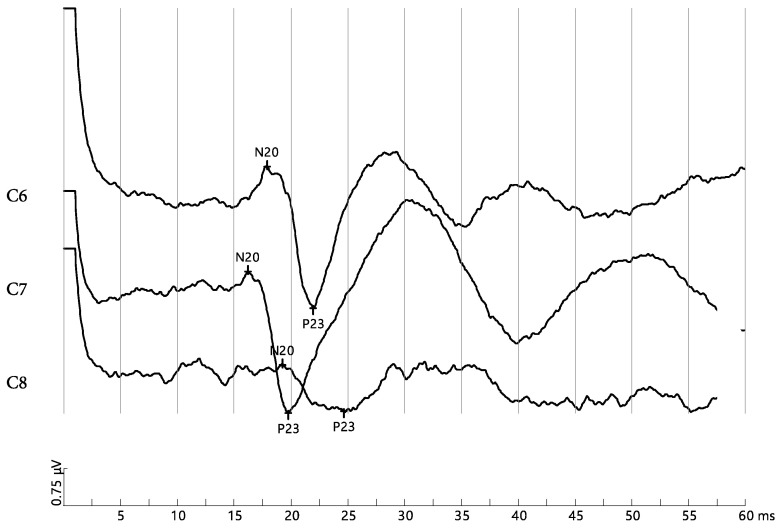
Short latency somatosensory-evoked potential of normal head posture (NHP) participant after prolonged slouched sitting for C6, C7, and C8. The amplitudes measured between N20 and P23 are: 2.81 μV, 2.81 μV, and 0.945 μV, respectively.

Following the prolonged sitting position of 30 min, the between-group statistical analysis was significantly different, showing a more favorable nerve root function in the slouched sitting position for the FHP group compared to the NHP group, while the erect sitting position demonstrated a significant favorability to the NHP group, as shown in Table 2. Figure 5 and Figure 6 show short latency DSSEPs for C6, C7 and C8 pre and post 30 min of sitting in a participant from the NHP group.

**Figure 7:**
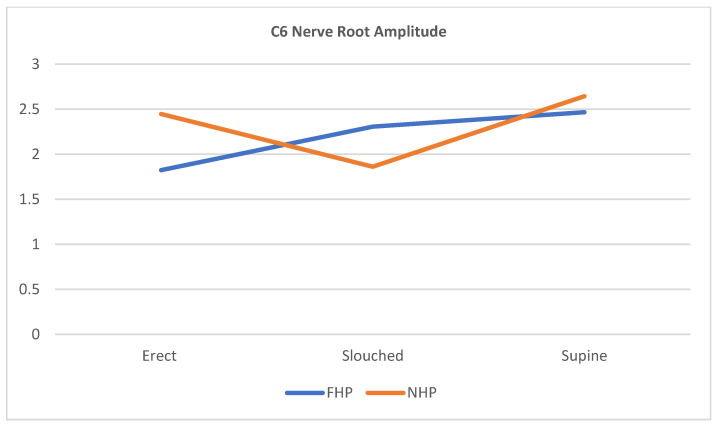
Scatterplot line of cervical nerve C6 amplitude relationship with the different sitting positions for participants with forward head posture (FHP) and normal head posture (NHP). The graph highlights that the FHP group has shown an increased amplitude during slouched sitting compared to erect sitting. The supine position shows the highest nerve peak from all three positions.

**Figure 8:**
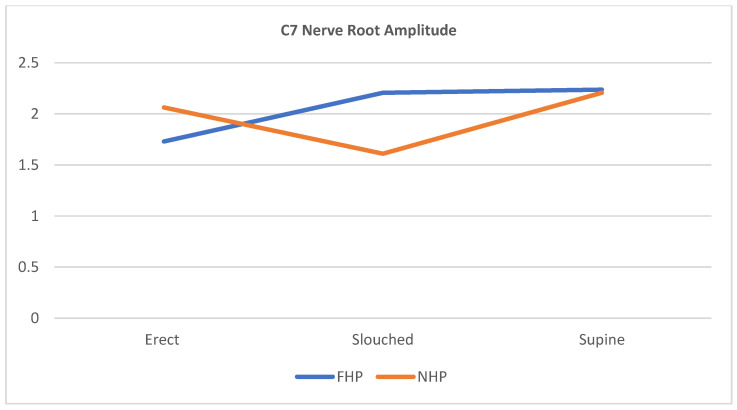
Scatterplot line of cervical nerve C7 amplitude relationship with the different sitting positions for participants with forward head posture (FHP) and normal head posture (NHP). The graph highlights that the FHP group has shown an increased amplitude during slouched sitting compared to erect sitting. The supine position shows the highest nerve peak from all three positions.

**Figure 9:**
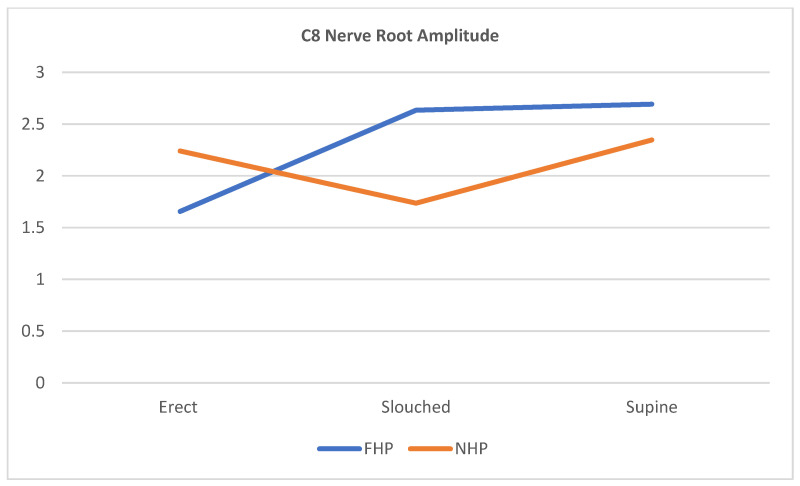
Scatterplot line of cervical nerve C8 amplitude relationship with the different sitting positions for participants with forward head posture (FHP) and normal head posture (NHP). The graph highlights that the FHP group has shown an increased amplitude during slouched sitting compared to erect sitting. The supine position shows the highest nerve peak from all three positions.

The scatterplots in Figure 7, Figure 8 and Figure 9 show that for all three cervical nerve roots (C6, C7, C8), their amplitudes increased in the slouched position for the FHP group compared to the erect position. Contrarily, the NHP group displayed a higher amplitude in the erect position than the slouched position. Both groups showed similarity in the nerve root functions in the prolonged supine position.

Simple main effects analysis showed that the head posture had a statistically significant effect on the cervical nerve root functions of C6 (*p* = 0.030), C7 (*p* = 0.025), and C8 (*p* < 0.001). As for the sitting posture, a statistical significance was also detected on the cervical nerve roots C6 (*p* < 0.001), C7 (*p* = 0.025), and C8 (*p* < 0.001). Analysis with Levene’s test of equality of error variances showed that the homogeneity of variances in our data can be assumed for C6 (*p* = 0.235), for C7 (*p* = 0.02), and for C8 (*p* = 0.068).

## 4. Discussion

As we had initially hypothesized, the cervical nerve root DSSEPs were identified to have significant differences between each of the positions tested: erect sitting, slouched sitting, and lying supine. Interestingly, our intergroup results (NHP vs. FHP groups) showed a pattern contrary to popular belief. The NHP group displayed the greatest peaks for DSSEPs while in the erect sitting position, and this is generally consistent with the previous literature on ideal sitting posture; namely, that altered cervical posture has damaging effects. In contrast, the individuals in the FHP group had the greatest peak-to-peak amplitude of DSSEPs while in the slouched position as opposed to the erect position. While the erect position is deemed the most correct and healthy position for the spine, our results show otherwise relative to the initial posture of the participant. Thus, our findings indicate the importance of considering the initial presenting cervical sagittal alignment of the individual as a significant factor when determining the ideal sitting posture. To our knowledge, this is the first research investigation that considers the cervical sagittal alignment as a contributing factor when assessing different sitting postures. These findings give new insights into an essential consensus of sitting that seem to suggest the uniqueness of the individual’s alignment. In other words, what works well for one person may create discomfort for another. Our main findings are in agreement with that of Dunk et al. who reported that individuals may respond differently to various sitting postures and the variables that influence sitting posture are still not fully understood [31]. Similarly, Adams suggested that sustained postures, including the erect posture if maintained for a prolonged period, can lead to discomfort and even injury [32].

One of the most important findings in this study was that for participants who already had FHP, adopting the erect sitting position negatively affected their nerve root function, as manifested by significant reductions in the peak-to-peak amplitude of the DSSEPs for the nerve roots tested. Some authors have noted that an erect sitting posture [14,15] may lead to increased levels of fatigue resulting from increased muscle activation compared with the habitual sitting posture of an individual. In contrast, Nishikawa et al. [18] identified that FHP compared to NHP was associated with a greater cervical spine muscle activity and subjective fatigue using high density surface EMG. These seemingly contradictory findings are challenging to explain and likely involve complex interactions between an individual’s perception of their natural posture, specific spine geometric alignments of the sagittal plane curvatures, muscle length tension relationships, and yet-undetermined variables.

It has been reported that FHP is associated with the weakening of isometric strength and endurance of the deep neck flexors [33]. The endurance of the deep neck flexor muscles directly affects the function of the cervical spine, and the strength of these muscles are important in maintaining the posture and stability of the neck [33,34,35]. Along with the shoulder girdle muscles, the deep neck flexors are crucial for the control and support of the neck, supporting the weight of the head against gravity and stabilizing the head [36]. Accordingly, it is expected that assuming the erect posture for people with FHP will induce more fatigue. Due to this, it is believed that FHP participants will be more comfortable if they adopt a slouched posture while relying on passive structures of the spine (ligaments and bone). During a slouched or slumped posture, it is proposed that this posture relies mainly on the passive (e.g., spinal ligaments) structures to maintain a resting sitting position. This results in a diminished requirement for muscle activity [37,38].

Related research has shown that muscle fatigue occurs when erect postures (such as upright sitting) are sustained for as little as 30 min, even if contractions are as low as 2% to 5% of the maximum voluntary contraction [39]. This offers a possible explanation as to why participants might prefer a slumped sitting posture—because it is perceived as less physically demanding [37,38]. Still, it is necessary to note that the decline in stabilizing potential of the paraspinal muscles, the associated compensatory antagonistic coactivation, and the related increase in spinal load are associated with muscle fatigue. As documented in many studies, fatigue-related changes in muscle stiffness may reduce the capacity of the paraspinal muscles to stabilize the spine. If fatigue is not severe (as expected in our study), then the compensatory recruitment of antagonistic co-contraction may restore stability, but this will contribute to increased spinal load and an associated risk of overload injury [40,41,42]. This aberrant spinal load caused by muscular fatigue might be a possible explanation for the decrease in the peak-to-peak amplitude of DSSEPs.

A final explanation for the reduced amplitude of the DSSEPs being different in the NHP vs. FHP groups during different sitting positions could be the amount and distribution of the cervical lordotic curve in the participants. It is known that abnormal cervical sagittal alignment (kyphosis, s-curves, etc.) creates changes in loading on the vertebrae and soft tissues [43]. Gong et al. [33] reported that reduced and kyphotic cervical curves coupled with FHP reduced the endurance of the deep neck flexors. Since it is known that increased FHP causes flexion of the lower cervical spine and extension of the upper cervical spine [44], it could be that slumped sitting in already FHP individuals causes a more dramatic increase in the lower cervical spine due to the increased thoracic kyphosis that also occurs in this posture. The increased cervical lordosis in this specific ‘exaggerated’ postural position might reduce the net tension on the lower cervical spinal cord and nerve roots, leading to an increased amplitude of the DSSEPs [20,45]. Though speculative, this seems like a plausible explanation that needs to be confirmed in future investigations using spine imaging.

### Study Limitations and Suggestions for Future Research

The following limitations should be considered when interpreting the current study’s findings. We only examined the lower cervical spine nerve roots C5, C6 and C7, without looking at other cervical levels. Additionally, participants in this study were young adults, and as result, the findings might not be applicable to other age groups. Given the limitations of the current study, future research is needed to analyze the other cervical nerve roots, to shed more light on the upper cervical region related to different sitting postures. Investigating the effects of different sitting postures in different age groups may also help researchers in understanding the function of age as a contributing factor. Lastly, we did not specifically investigate the smoking status of a participant as an independent variable herein. However, the fact that there were almost an equal number of smokers in the two groups eliminated the possibility that smoking could have an impact on the outcome measure as a confounding variable between our two groups, as was shown. Still, we suggest that future research should take smoking status into consideration. Finally, our investigation did not formally investigate the true ideal geometric sitting posture of the thoracic and thoraco-lumbar pelvic region, nor did it investigate mechanisms for attaining or improving altered posture positions in participants, as has been performed in previous investigations [46,47]. Future work could incorporate the key findings herein of how the CVA of an individual affects nerve root function in different sitting positions and how variations in ideal sitting postures and its training or re-training are affected.

## 5. Conclusions

We identified statistically significant differences in the cervical nerve root function in all postures between the NHP and FHP groups (*p* < 0.001), indicating that the FHP and NHP reacted differently in different positions. For the supine reference position, we found no significant differences between the FHP and NHP groups for the DSSEPs of nerve roots C6–C8. In contrast, both the erect and slouched sitting positions were found to have significant differences in nerve root amplitudes between the NHP and FHP groups. Specifically, the NHP group was found to have the greatest peaks for nerve root DSSEPs while in the erect sitting position and this is generally consistent with the previous literature on ideal sitting posture; namely, that altered cervical posture has damaging effects in sitting posture. However, the participants in the FHP group demonstrated the largest peak-to-peak amplitude of DSSEPs for nerve roots C6–C8 while in the slouched position as opposed to an erect position. The ideal sitting posture and its influence on cervical nerve root function may be dependent upon the underlying initial forward head posture presentation of a person, however, further research is needed to corroborate these findings in patients with and without cervical spine disorders.

## Figures and Tables

**Table 1:** Participant demographic variables listed as means and standard deviations. There were no statistically significant differences between the NHP and FHP groups; *p* > 0.05 for all variables, using the independent t-test for continuous data and chi-squared test of independence for categorical data.

Characteristic	Forward Head Group (n = 30)	Normal Head Group (n = 30)	Significance
Age (years)	20.5 ± 2	20 ± 3	0.4
Weight (kg)	61.2 ± 4	62.2 ± 5	0.3
BMI	18.4 ± 1.2	18.3 ± 1.4	0.7
Smoking			
Nonsmoker	15	14	0.6
Light Smoker	10	12	
Heavy smoker	5	4	
Sex			
Male	11	11	-
Female	19	19	
CVA	41.7 ± 2	66.9 ± 4.6	<0.001

**Table 2:** Two-way analysis of variance results. FHP = forward head posture group, NHP = neutral head posture group, Erect = neutral upright sitting posture, Slouched = slouched or slumped sitting posture, Supine = lying supine analysis, C6, C7, C8 = the respective nerve roots tested, C.I. = confidence interval.

		Erect	Slouched	Supine
C6	FHP	1.84 ± 0.33	2.30 ± 0.38	2.50 ± 0.37
	NHP	2.51 ± 0.36	1.92 ± 0.30	2.60 ± 0.42
*p*-value	*p* < 0.001		*p* < 0.001	*p* = 0.09
C.I.	[−0.80, −0.44]		[0.27, 0.62]	[−0.38, 0.03]
C7	FHP	1.71 ± 0.23	2.24 ± 0.15	2.22 ± 0.20
	NHP	2.11 ± 0.38	1.60 ± 0.25	2.22 ± 0.42
*p*-value	*p* = 0.001		*p* < 0.001	*p* = 0.72
C.I.	[−0.50, −0.17]		[0.49, 0.70]	[−0.14, 0.20]
C8	FHP	1.71 ± 0.41	2.61 ± 0.56	2.73 ± 0.55
	NHP	2.21 ± 0.40	1.70 ± 0.31	2.34 ± 0.46
*p*-value	*p* < 0.001		*p* < 0.001	*p* = 0.01
C.I.	[−0.80, −0.38]		[0.66, 1.13]	[0.08, 0.61]

**Table 3:** Two-way analysis of variance results.

Tests of Between-Subjects Effects						
	Type III Sum of Squares	df	Mean Square	F	Sig.	Partial Eta Squared
C6 amplitude						
Corrected Model	17.272	5	3.454	26.294	<0.001	0.430
Intercept	917.742	1	917.742	6985.400	<0.001	0.976
Head Posture	0.629	1	0.629	4.787	0.030	0.027
Sitting	8.007	2	4.004	30.474	<0.001	0.259
Head Posture * Sitting	8.636	2	4.318	32.867	<0.001	0.274
Error	22.860	174	0.131			
C7 amplitude						
Corrected Model	11.095	5	2.219	26.217	<0.001	0.430
Intercept	726.374	1	726.374	8581.961	<0.001	0.980
Head Posture	0.431	1	0.431	5.095	0.025	0.028
Sitting	4.075	2	2.037	24.070	<0.001	0.217
Head Posture * Sitting	6.589	2	3.295	38.926	<0.001	0.309
Error	14.727	174	0.085			
C8 amplitude						
Corrected Model	28.892	5	5.778	27.731	<0.001	0.443
Intercept	885.470	1	885.470	4249.485	<0.001	0.961
Head Posture	2.185	1	2.185	10.484	0.001	0.057
Sitting	9.892	2	4.946	23.737	<0.001	0.214
Head Posture * Sitting	16.815	2	8.407	40.348	<0.001	0.317
Error	36.257	174	0.208			

**Table 4:** Pairwise comparisons.

(I) Sitting	(J) Sitting	Mean Difference (I–J)	Std. Error	Sig. b	95% Confidence Interval for	
					Difference b	
					Lower Bound	Upper Bound
Dependent Variable: C6 Amplitude						
Erect	Slouched	0.051	0.066	1	−0.109	0.211
	Supine	−0.420 *	0.066	<0.001	−0.580	−0.260
Dependent Variable: C7 Amplitude						
Erect	Slouched	−0.237 *	0.083	0.015	−0.439	−0.036
	Supine	−0.572 *	0.083	<0.001	−0.773	−0.370
Dependent Variable: C8 Amplitude						
Erect	Slouched	−0.012	0.053	1	−0.140	0.117
	Supine	−0.325 *	0.053	<0.001	−0.453	−0.196

* The mean difference is significant at the 0.05 level. b. Adjustment for multiple comparisons: Bonferroni.

## Data Availability

The datasets analyzed in the current study are available from the corresponding author on reasonable request.
